# Diffusion Tensor Imaging and Chemical Exchange Saturation Transfer MRI Evaluation on the Long-Term Effects of Pulsed Focused Ultrasound and Microbubbles Blood Brain Barrier Opening in the Rat

**DOI:** 10.3389/fnins.2020.00908

**Published:** 2020-08-25

**Authors:** Tsang-Wei Tu, Zsofia I. Kovacs, Maggie Sundby, Jaclyn A. Witko, Georgios Z. Papadakis, William C. Reid, Dima A. Hammoud, Joseph A. Frank

**Affiliations:** ^1^Molecular Imaging Laboratory, Department of Radiology, Howard University College of Medicine, Washington, DC, United States; ^2^Frank Laboratory, Radiology and Imaging Sciences, Clinical Center, National Institutes of Health (NIH), Bethesda, MD, United States; ^3^Center for Neuroscience and Regenerative Medicine, Henry Jackson Foundation, Bethesda, MD, United States; ^4^Institute for Biomedical Engineering, Swiss Federal Institute of Technology, Zurich, Switzerland; ^5^Center for Infectious Disease Imaging, Clinical Center, National Institutes of Health (NIH), Bethesda, MD, United States; ^6^National Institute of Biomedical Imaging and Bioengineering, National Institutes of Health (NIH), Bethesda, MD, United States

**Keywords:** pFUS microbubble, blood brain barrier, T2^∗^ abnormality, DTI, CEST, FDG-PET

## Abstract

Blood-brain barrier opening (BBBO) with pulsed Focused Ultrasound (pFUS) and microbubbles (MB) has received increasing interest as a method for neurotherapeutics of the central nervous system. In general, conventional MRI [i.e., T2w, T2^∗^w, gadolinium (Gd) enhanced T1w] is used to monitor the effects of pFUS+MB on BBBO and/or assess whether sonication results in parenchymal damage. This study employed multimodal MRI techniques and ^18^F-Fludeoxyglucose (FDG) PET to evaluate the effects of single and multiple weekly pFUS+MB sessions on morphology and glucose utilization levels in the rat cortex and hippocampus. pFUS was performed with 0.548 MHz transducer with a slow infusion over 1 min of Optison^TM^ (5–8 × 10^7^ MB) in nine focal points in cortex and four in hippocampus. During pFUS+MB treatment, Gd-T1w was performed at 3 T to confirm BBBO, along with subsequent T2w, T2^∗^w, DTI and glucose CEST (glucoCEST)-weighted imaging by high field 9.4 T and compared with FDG-PET and immunohistochemistry. Animals receiving a single pFUS+MB exhibited minimal hypointense voxels on T2^∗^w. Brains receiving multiple pFUS+MB treatments demonstrated persistent T2w and T2^∗^ abnormalities associated with changes in DTI and glucoCEST when compared to contralateral parenchyma. Decreased glucoCEST contrast was substantiated by FDG-PET in cortex following multiple sonications. Immunohistochemistry showed significantly dilated vessels and decreased neuronal glucose transporter (GLUT3) expression in sonicated cortex and hippocampus without changes in neuronal counts. These results suggest the importance to standardize MRI protocols in concert with advanced imaging techniques when evaluating long term effects of pFUS+MB BBBO in clinical trials for neurological diseases.

## Introduction

MRI guided (MRIg) pulsed Focused Ultrasound (pFUS) is a non-invasive technique being advocated for the blood brain barrier opening (BBBO) to facilitate the delivery of neurotherapeutics (i.e., drugs, genes, biologics) in the treatment of primary and metastatic central nervous system (CNS) tumors (Park et al., 2012, 2020) or neurodegenerative diseases, such as amyotrophic lateral sclerosis (Abrahao et al., 2019) and Alzheimer’s disease (Baseri et al., 2012; Kovacs et al., 2014; Lipsman et al., 2018). pFUS coupled with an infusion of ultrasound contrast agent microbubbles (MB) causes BBBO primarily by mechanical effects from acoustic cavitation forces on the endothelium that alters the integrity of tight junction proteins (TJP) and changes calcium fluxes within vasculature (Sheikov et al., 2004, 2008; Deng, 2010; Burgess and Hynynen, 2014; Gorick et al., 2020). MRIg pFUS+MB targeting delivery of neurotherapeutics in CNS diseases has been performed in preclinical experimental studies (Jones et al., 2018) and ongoing clinical trials (NCT03608553, NCT03739905, NCT04118764, NCT03551249, NCT03616860, NCT03714243)^1^. Aside from the positive results, the pFUS+MB BBBO has also been reported to induce a sterile inflammatory response (SIR) in the targeted parenchyma (Banks, 2016; Kovacs et al., 2017b, 2018b; McMahon and Hynynen, 2017; McMahon et al., 2017, 2020). The magnitude of the expression of cytokines, chemokines and trophic factors (CCTF) and cell adhesion molecules (CAM) may be related to the sonication parameters used along with MB dosing (Silburt et al., 2017; Kovacs et al., 2018a). The long-term effects of the induced SIR in pFUS+MB BBBO requires further investigation.

MRI is a sensitive and commonly used technique to assess changes in the brain following pFUS+MB. Gadolinium-based contrast agents (GBCA)-T1-weighted (w) MRI are used to document BBBO following sonication, confirmed by extravasation of dyes or plasma proteins into the parenchyma on histology (Yang et al., 2009; Chai et al., 2018; Stavarache et al., 2018; Morse et al., 2019). The level of contrast enhancement in GBCA-T1w MRI may be related to the magnitude of sonication parameters and the amount of CCTF expression (Nance et al., 2014; Mooney et al., 2016; Olumolade et al., 2016; Kovacs et al., 2017b; McMahon et al., 2017). T2w (Alkins et al., 2013; Aryal et al., 2015; Meng et al., 2017) and T2^∗^w (Hynynen et al., 2001) images have also been used to examine the structural damage and microhemorrhages following pFUS induced BBBO (Gan et al., 2012; McDannold et al., 2012; Kovacs et al., 2014; Downs et al., 2015b; Alecou et al., 2017; Horodyckid et al., 2017). In addition, immunohistochemistry (IHC) techniques have also been used to document changes in cellular activation following sonication and BBBO (Hynynen et al., 2001; Aryal et al., 2015). However, investigations using advanced high-resolution imaging techniques that can assess changes in tissue morphology and glucose utilization, such as diffusion tensor imaging (DTI) (Basser and Pierpaoli, 1996) and chemical exchange saturation transfer (CEST) (Ward et al., 2000). MRI have received little attention despite their potential for assessing treatment effects following single or multiple sonication sessions.

The purpose of this study was to evaluate the long-term effects of single and multiple pFUS+MB BBBO in the rat cortex and hippocampus by high-resolution advanced MRI techniques and correlate radiological findings to pathology. MRI was performed at 9.4 T at 1 day, 2 and 6 weeks post 1×, 2×, and 6× pFUS+MB using T2w, T2^∗^w, DTI and CEST imaging. The imaging results were compared to 2-[^18^F] Fluoro-deoxyglucose (FDG) positron emission tomography (PET) performed after the sixth sonication and IHC results for glucose transporters and neuronal density. The results underscore the value of incorporating advanced MRI imaging techniques in assessing metabolic and morphological changes in the brain following sonication.

## Materials and Methods

### Animal Care

For all the animal experiments, barrier-raised and specific pathogen-free 6–8-week-old female Sprague-Dawley rats were used (Charles River Laboratory, Wilmington, MA). Rats were housed individually in a temperature- and light-controlled room on a 12-h light–dark cycle and were fed commercial rodent chow (2018 Teklad Global 18% Protein Rodent Diet; Harlan Laboratories Inc., Indianapolis, IN) and tap water *ad libitum*. This study was approved by our Institutional Animal Care and Use Committee and all experiments were complied with the National Research Council’s Guide for the Care and Use of Laboratory Animals (2011).

### Experiment Design

**FIGURE 1:**
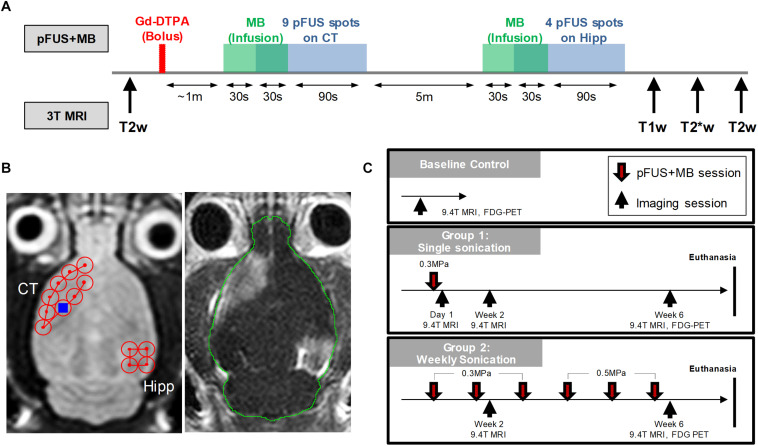
Experimental design. **(A)** Workflow of the MRI-guided pFUS+MB treatments on 3 T MRI system. **(B)** Axial T2w images (left) were acquired for sonication planning of the treatment: nine 2 mm-diameter non-overlapping focal points were placed on the left cortex covering the area anterior to the lateral ventricle and four focal points were placed on the right hippocampus. After bolus Gd-DTPA injection, microbubbles were slowly infused via tail and followed by sonication starting 30 s after microbubble infusion. The total sonication time is 120 s for each treatment. Gd-T1w images (right) shows the opening of blood-brain barrier after pFUS+MB treatment. **(C)** Longitudinal imaging experiment timeline. Baseline T2w MRI were first obtained on 9.4 T MRI to rule out brain abnormalities prior to sonication. The other imaging protocols included T2*w, DTI, CEST and ^18^F-FDG-PET. Animals were then euthanized in week 7 and brains were harvested for histological examination.

Figure 1 is a diagram outlining the timing of the imaging experiments performed. Rats had *in vivo* MRI using a Bruker 9.4T scanner (Bruker Corp., Billerica, MA) and a radiofrequency quadrature coil (Doty Scientific, Inc., Columbia, SC). Before starting pFUS+MB treatments, anatomical T2w imaging was used to screen for baseline brain abnormalities, including spontaneous ventriculomegaly previously found in the Wistar rats (Tu et al., 2014), which served as exclusion criteria. The imaging parameters included: rapid acquisition with refocused echoes (RARE) sequence, repetition time/echo time (TR/TE) 3000/11 ms, RARE factor 8, in-plane resolution 100 × 100 μm^2^, slice thickness 500 μm, slice number 30. Throughout the MRI scans, a circulating water warming pad was placed under the animals to ensure a constant 37°C body temperature while anesthesia was ensured by isoflurane (1–1.5%) in 100% O_2_ via nosecone. A steady respiratory rate was monitored using a pressure sensor (SA Instruments Inc., Stony Brook, NY) and maintained at 40–50 breaths-per-minute by controlling the level of isoflurane/oxygen mixture. 21 rats were first scanned to screen for baseline abnormalities, 14 rats were determined to be normal, and 10 of these rats received further complete imaging examinations to serve as the baseline controls due to the limitation on the imaging resources. These 14 rats were randomly divided into two groups receiving either one (Group 1, *n* = 7) or 6 weekly (Group 2, *n* = 7) pFUS+MB treatments (Figure 1). After receiving one pFUS+MB exposures, Group 1 rats had 9.4 T MRI performed on week 2 (1X-W6) and week 6 (1X-W6) post-sonication and were euthanized at week 7. Group 2 rats received 6 weekly pFUS+MB treatments and had MRI at week 2 (2X-W2) and week 6 (6X-W6) post-sonication, and were euthanized at week 7 (Figure 1C). ^18^F-FDG-PET scans were performed on week 6 on a randomly selected subsets of control (*n* = 4), Group 1 (*n* = 6) and Group 2 (*n* = 4) rats. The imaging results presented in the current study were complementary to the data reported in Kovacs et al. (2018b).

### MRI-Guided Pulsed Focused Ultrasound and Microbubbles

MRI-guided pFUS+MB treatments were performed using a preclinical pFUS instrument equipped with a surface coil (RK-100, FUS Instruments, Toronto, ON) on a Philips Achieva 3 T MRI system (Philips Healthcare, Andover, MA). The workflow of the pFUS+MB treatments is illustrated in Figure 1A. pFUS targeting coordinates for sonication were obtained from axial T2w images by turbo spin echo (TSE) with TR/TE 2000/70 ms, echo train length 12, in-plane resolution 273 × 273 μm^2^, slice thickness 1.5 mm, average 4. Prior to pFUS sonication, rats were first infused intravenously (IV) with 100 μL Magnevist gadopentetate dimeglumine (Gd, Bayer Healthcare Pharmaceuticals, Inc., Whippany, NJ) via tail vein catheter for Gd-T1w MRI examination of BBBO. Thirty seconds before initiating sonication, an intravenous infusion (1.66 μL/s) of Optison^TM^ (GE Healthcare, Little Chalfont, Buckinghamshire, United Kingdom) was started that continued to 100 μL (i.e., 30 s) during sonication to targeted regions in the left cortex and the right hippocampus with non-overlapping 2-mm diameter focal spots as previously described (Figure 1B; Kovacs et al., 2017b, 2018a,b). Infusion of MB was separated by at least 5 min between sonications in the same rat. The initial dose (Day 0) of Optison^TM^ was ∼460 μL/kg and was fixed at 100 μL (5–8 × 10^7^ MB) for all sonications independent of animal weight that was sufficient to cause BBBO (Kovacs et al., 2017b; Kovacs et al., 2018a). Immediately post-pFUS+MB, axial T1w images were obtained by spin echo (SE, TR/TE 215/10 ms, in-plane resolution 137 × 137 μm^2^, slice thickness 1.5 mm, average 4). Pulsed FUS was performed with the following parameters: 0.3–0.5 MPa peak negative pressure (PNP) measured in water that was applied in 10 ms burst length and <1% duty cycle with a pulse repetition frequency (PRF) of ∼0.5–0.6 Hz (i.e., 120 s/9 focal points in the left frontal cortex anterior to the lateral ventricle including the striatum, 120 s/4 target points in the right hippocampus (Figure 1B), using a single-element spherical FUS transducer (center frequency: 548 kHz focal number: 0.8 active diameter: 7.5 cm; FUS Instruments, Toronto, Canada). For Group 2 rats the PNP was increased to 0.5 MPa from the 4th sonication due to the changes in skull thickness and weight gain causing an effective decrease in MB/kg concentration in the animals. The pFUS PNP of 0.3–0.5 MPa were chosen while the animals were on 100% O_2_ that would result in detectible contrast enhancement on Gd-T1w images (McDannold et al., 2017; Kovacs et al., 2018b).

### *In vivo* 9.4 T MRI for pFUS+MB Treatment Effects

High-resolution 9.4 T MRI scans were obtained in the rat brain for the longitudinal examinations after pFUS+MB exposures as outlined in Figure 1C. The 9.4 T MRI scans were performed within 3 days after sonication using the following imaging protocols: T2w images by RARE (TR/TE 3000/11.6 ms; RARE factor 8; in-plane resolution 200 × 200 μm^2^; slice thickness 500 μm; average 3); three dimensional T2^∗^w images by multiple gradient echo (MGE; TR/TE 50/3 ms; ΔTE 3 ms; number of echoes 10; flip angle 30°; resolution 200 μm^3^ isotropic; average 2). The T2^∗^w images were generated by combining the 10 MGE data with an effective TE 22.7 ms to enhance the presence of T2^∗^ abnormality. Quantitative T2^∗^ maps were obtained by fitting the signal intensity (S_i_) of each voxel from MGE dataset to a mono-exponential decay as a function of TE:

(1) S=iSexp0(-TE/iT2*)

Three dimensional DTI was acquired using spin echo (SE) echo-planar imaging (EPI; TR/TE 550/49 ms; EPI segment 6; Δ 15 ms; δ 5 ms; b-value 800 s/mm^2^; 15 diffusion encoding directions with 2 B_0_ images; average 1). The DTI imaging volume and voxel size were identical to the T2^∗^w imaging. Diffusion-weighted images were corrected for B_0_ susceptibility induced EPI distortion, eddy current distortions, and motion distortion with b-matrix reorientation using Tortoise v2.5 (NIH, Bethesda, MD). After correction, the diffusion tensor was calculated to derived DTI parameters, fractional anisotropy (FA), mean diffusivity (MD), axial diffusivity (AD), and radial diffusivity (RD) to evaluate the microstructural changes following pFUS+MB opening of the BBB. CEST MRI data were acquired by single slice RARE with (S_MT_) and without (S_0_) magnetization transfer (MT) pulses (TR/TE 3091.7/10.4 ms; RARE factor 8; in-plane resolution 200 × 200 μm^2^; slice thickness 0.8 mm; MT pulse 1.5 μT, 1 s). The MT offset frequencies (Δω) were set from −1.6 to +1.6 kHz with 100 Hz stepping. Z-spectral interpolation and WAter Saturation Shift Referencing (WASSR) techniques were applied to correct the shifted water resonance frequency by B_0_ inhomogeneity (TR/TE 1500/10.4 ms; RARE factor 8; MT pulse 0.3 μT, 250 ms; Δω−0.4 to +0.4 kHz with 60 Hz stepping). Magnetization transfer ratio asymmetry (MTR_asym_) was calculated by [S_MT_(−Δω) − S_MT_(Δω)]/S_0_. The CEST-weighted images were generated by integrating the area under the curves of MTR_asym_ for the proton signal of glucose metabolites (glucoCEST) exchanging at the center of 1.2 ppm (±0.5 ppm) in arbitrary unit (Van Zijl et al., 2007; Chan et al., 2012; Tu et al., 2018). One Group 2 animal had severe motion artifacts in the week 6 MRI scans. This dataset was excluded in the rest of analysis.

### *In vivo* ^18^F-FDG-PET

^18^F-FDG-PET imaging was performed at week 6 for each group to evaluate the changes of glucose uptake and metabolism following pFUS+MB treatment using a small animal Inveon PET/CT scanner (Siemens Medical Solutions, Malvern, PA) with transaxial field of view (FOV) of 10 cm, axial FOV of 12.7 cm and full width at half maximum (FWHM) spatial resolution at center FOV of 1.4 mm. Rats were first anesthetized with 2–2.5% isoflurane-oxygen mixture and ^18^F-FDG (dose based on the body weight of each animal; avg. dose = 0.951 mCi) was then injected as a bolus through the tail vein followed by a quick saline flush (300 μL). After the injection, the animals were allowed to recover at room temperature. Thirty minutes after radiotracer injection, the animals were anesthetized again and secured to an imaging bed, placing the head symmetrically in the center FOV. The animals’ respiratory rate was carefully monitored to avoid any intra-subject variability of the anesthesia level (target respiratory frequency range = 40–60 breaths/min). The body temperature was maintained by a heating pad. Following a 30 min uptake period, PET emission scans were acquired in list mode following which emission sinograms were corrected for scatter, ^18^F-decay, random and dead time. The resulting histograms were then reconstructed applying Fourier rebinning and ordered subject expectation maximization algorithm (4 OSEM iterations, 18 MAP iterations, matrix: 128 × 128, target resolution: 0.8 mm). Once scan was completed, the animals were allowed to recover from anesthesia under a heat lamp. The PET data were co-registered to a previously acquired T2w anatomical MR template and the standardized uptake values (SUVs) were processed using PMOD 3.7 (PMOD technologies, Zurich, Switzerland).

For all the MRI and PET data quantification, the regions of interest (ROIs) encompassing the ipsilateral treated and contralateral untreated regions were drawn for cortex and hippocampus by two experienced technologists (Supplementary Figure S1). ROIs for DTI and T2^∗^ maps also included external capsule (EC) to assess white matter integrity after pFUS+MB. Except for the aforementioned software, the imaging data were processed by in-house Matlab (Mathworks, Inc., Natick, MA) scripts.

### Immunohistochemistry Analysis

Three animals in each group were randomly selected at week 7 after last scan for trans-cardiac perfusion with 4% PFA in PBS. The brains were extracted and sectioned at 10 μm for histological examination for neurons and glucose transporters per published protocol (Yu et al., 1995; Kovacs et al., 2018b). The following primary antibodies were used: glucose transporter 1 (GLUT1) (Invitrogen MA5-11315, Waltham, MA) at 1/200; glucose transporter 3 (GLUT3) (Abcam ab41525, Cambridge, United Kingdom) at 1/1500; hexaribonucleotide binding protein-3 (NeuN) (Cat. mab377, Millipore) at 1/1000. Secondary antibodies were used at 1/200 as follows: NeuN: goat anti-mouse F(ab’) IgG- H&L Dylight 594 (Cat. ab96881, Abcam, Cambridge, United Kingdom); GLUT1: goat anti-mouse F(ab’) IgG2a Dylight 594 (SAB4600328, Sigma Aldrich, St. Louis, MO); GLUT3: goat anti-rabbit F(ab’) IgG- H&L Dylight 594 (Cat. ab102293, Abcam, Cambridge, United Kingdom). The IHC data were quantified from the corresponding locations matching to the ROIs for MRI data quantification to cover each pFUS treated and contralateral regions. In each IHC images, the positive fluorescent staining was quantified from three FOVs in 20X and averaged to represent the area percentage of the staining.

### Statistical Analysis

Statistical analysis was performed using Prism v8.1.2 (GraphPad Software, Inc., La Jolla, CA). The experiment was powered around the MRI variables, including T2^∗^, DTI, and CEST parameters. A sample size of *n* = 6 –10/group was determined necessary to detect differences in these key variables at an α level of *p* < 0.05 and 80% power. The quantification data of the T2^∗^ and DTI parameters passed the Shapiro–Wilk test for normality and were compared between the unsonicated animals of the baseline control, animals sonicated once and imaged at week 6 (1X-W6), and animals sonicated six times and imaged at week 6 (6X-W6) using paired *t*-test in significant level predetermined at *p* < 0.05. For glucoCEST and FDG-PET, the contrast ratios between the ipsilateral treated and contralateral untreated brain regions were calculated and reported for the cortex and hippocampus. Because of the non-normality of measured variables distribution, the nonparametric Kruskal–Wallis test followed by Dunn’s *post-hoc* multiple comparisons test (*p* < 0.05) was used to compare the glucoCEST and FDG-PET contrast ratios among groups. The comparison included unsonicated animals of the baseline control, animals sonicated once and imaged at week 6 (1X-W6), and animals sonicated six times and imaged at week 6 (6X-W6). A separate longitudinal analysis was performed for Group 1 rats between glucoCEST ratios at 2 weeks (1X-W2) and at 6 weeks (1X-W6), and for Group 2 rats at week 2 (2X-W6) and at week 6 (6X-W6) scans. The IHC data passed normality test and compared using paired *t*-test between the ipsilateral treated and contralateral untreated regions. All data are reported as mean ± SD.

## Results

### T2w, T2^∗^w, and DTI

**FIGURE 2:**
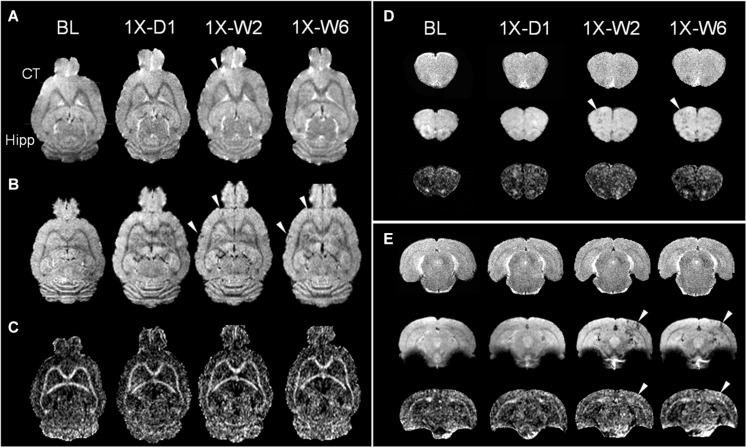
Longitudinal MRI of the brain in a single pFUS+MB treatment group (1X) acquired in baseline (BL), day 1 (D1), week 2 (W2), and week 6 (W6) by **(A)** T2w, **(B)** T2*w, and **(C)** fractional anisotropy (FA) of 9.4 T MRI. The T2*w images in week 2 and 6 display apparent T2* abnormalities (arrowheads) in **(B,D)** the left cortex and **(E)** right hippocampus, where DTI shows increased FA correspondingly. No abnormal voxel is found in the FA images in (C) and (D). The horizontal lines in **(A)** denote the coronal section in **(D,E)**. **(D,E)** Images from upper to lower are T2w, T2*w, FA. CT, cortex; Hipp, hippocampus.

**FIGURE 3:**
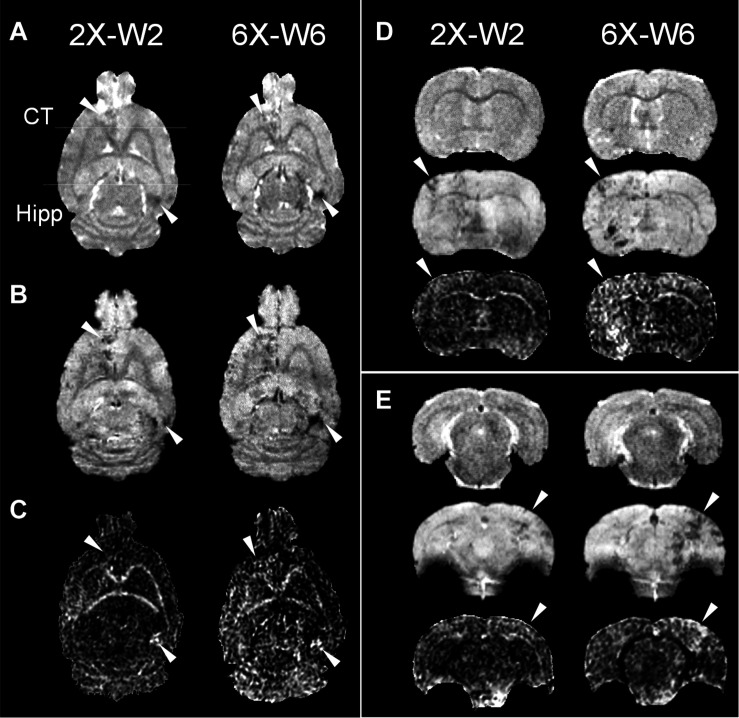
Longitudinal MRI of the brain in the weekly pFUS+MB treatment group (2X or 6X) imaged in week 2 (W2) and week 6 (W6) by 9.4 T **(A)** T2w, **(B)** T2*w, and **(C)** fractional anisotropy (FA). The T2w and T2*w images demonstrate clear treatment effects after multiple pFUS+MB treatments, where DTI clearly shows increased FA. The arrowheads indicate the locations that show abnormalities after treatments. The horizontal lines in (A) denote the coronal section in **(D,E)**. **(D,E)** Images from upper to lower are T2w, T2*w, FA. CT, cortex; Hipp, hippocampus.

**FIGURE 4:**
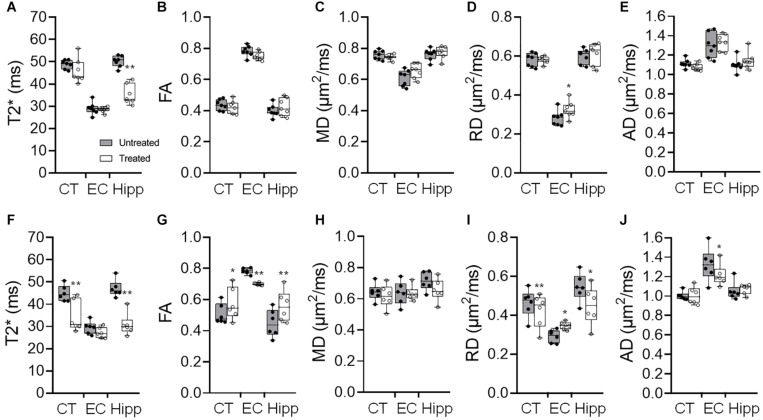
**(A–E)** Quantification of the 9.4 T imaging data acquired in week 6 brains of Group 1 rats with a single pFUS+MB sonication (1X-W6), and (F-J) Group 2 with six weekly sonications (6X-W6) of T2* **(A,F)**, fractional anisotropy (FA) **(B,G)**, mean diffusivity (MD) **(C,H)** and radial diffusivity (RD) **(D,I)** and axial diffusivity (AD) **(E,J)**. Comparing to the brains with one sonication, changes in the DTI parameters are more significant between the ipsilateral treated and contralateral untreated regions of the cortex (CT), external capsule (EC) and hippocampus (Hipp) after 6 weekly pFUS+MB treatments. *p < 0.05, **p < 0.01 vs. contralateral untreated region, paired *t*-test (Group 1, *n* = 7; Group 2, *n* = 6).

pFUS+MB exposure to the cortex and hippocampus resulted in significant contrast enhancement in Gd-T1w images indicative of BBBO (Figure 1B). In Group 1 rats, MRI demonstrated no qualitative differences between ipsilateral treated brain and contralateral parenchyma on T2w, T2^∗^w, and DTI images 1-day post-pFUS+MB (Figure 2). MRI scans performed at week 2 and week 6 post-sonication revealed scattered hypointense voxels on T2^∗^w images in the targeted cortex and hippocampus in five of seven rats (Figures 2B,D,E). DTI showed hyperintense voxels in the FA maps of the treated hippocampus (Figure 2E). In Group 2 rats that received 6 weekly pFUS+MB, there were clear differences after the second sonication on T2 and T2^∗^w images with hypointense voxels in the targeted cortex and hippocampus (Figure 3). The hyperintense voxels appeared more clearly in the FA maps of the treated cortex and hippocampus that were consistent with areas of abnormalities on T2^∗^w images (Figures 3C–E). Compared to the contralateral regions, quantitative analysis demonstrated significantly decreased T2^∗^ values in the cortex of the Group 2 rats (−23.71 ± 10.67%, *t*_(5)_ = 5.818, *p* < 0.01), and in the hippocampus in both Group 1 [−29.8 ± 9.1%, *t*_(__6)_ = 8.226, *p* < 0.001] and Group 2 [−32.98 ± 14.06%, *t*_(__5)_ = 6.780, *p* < 0.01] rats at week 6 (Figures 4A,F). In Group 1 rats, a significant increase in RD was observed in the treated EC [+15.89 ± 13.08%, *t*_(__6)_ = 3.582, *p* < 0.05] (Figure 4D). All the rest of the DTI metrics were not different between the treated and untreated hemisphere at week 6 after one sonication. In Group 2 rats, compared to the contralateral untreated regions, FA increased significantly in the treated cortex [+11.67 ± 9.2%, *t*_(__5)_ = 3.220, *p* < 0.05] and hippocampus [+25.69 ± 13.61%, *t*_(__5)_ = 5.852, *p* < 0.01], while RD decreased in the cortex [−9.16 ± 6.41%, *t*_(__5)_ = 4.151, *p* < 0.01] and hippocampus [−18.07 ± 13.25%, *t*_(__5)_ = 3.436, *p* < 0.05] at week 6, along with significantly decreased FA [−10.55 ± 3.15%, *t*_(__5)_ = 7.719, *p* < 0.001], increased RD [+19.68 ± 14.69%, *t*_(__5)_ = 3.658, *p* < 0.05], and decreased AD [−7.96 ± 5.12%, *t*_(__5)_ = 3.680, *p* < 0.05] in the EC (Figures 4G–J). The T2^∗^ and DTI results are also listed in the Supplementary Table S1.

### GlucoCEST Weighted Imaging

**FIGURE 5:**
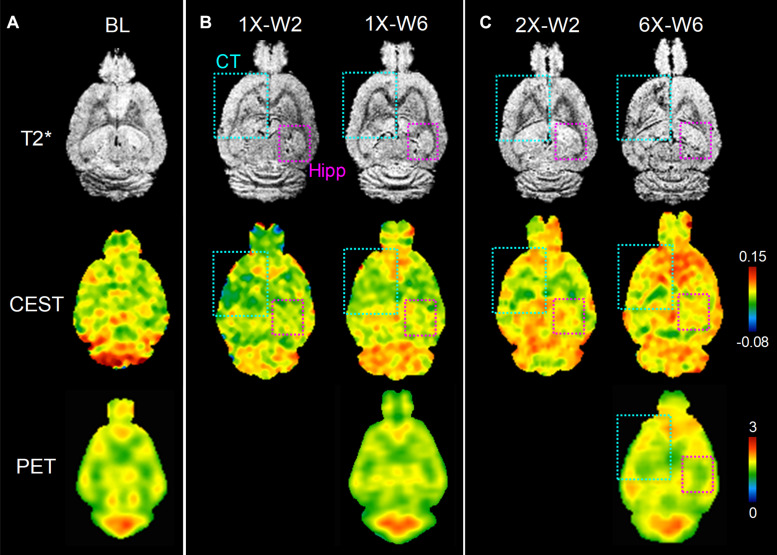
*In vivo* MRI and FDG-PET images of rat brains of **(A)** baseline (BL), **(B)** one sonication (1X), **(C)** multiple weekly pFUS+MB treatments (2X, 6X) and images at week 2 (W2) and week 6 (W6). T2*w images clearly show the sonication sites in the left cortex and striatum (cyan square), and in the right hippocampus (purple square), where the glucoCEST-weighted images and FDG-PET images show changes correspondingly.

**FIGURE 6:**
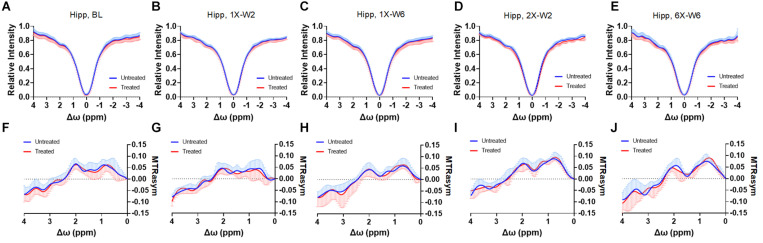
**(A–E)** The CEST Z-spectra and **(F–J)** MTR_asym_ of the treated and untreated hippocampus from baseline (**A,F**, *n* = 10), 1X-W2 (**B,G**, *n* = 7), 1X-W6 (**C,H**, *n* = 7), 2X-W2 (**D,I**, *n* = 7), 6X-W6 (**E,J**, *n* = 6) rats. Data are presented as mean ± SD.

**FIGURE 7:**
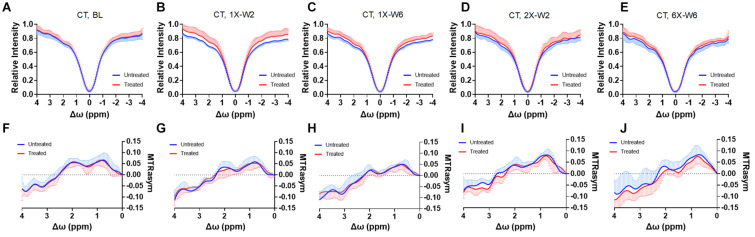
**(A–E)** The CEST Z-spectra and **(F–J)** MTR_asym_ of the treated and untreated cortex from baseline (A, F, *n* = 10), 1X-W2 (B, G, *n* = 7), 1X-W6 (C, H, *n* = 7), 2X-W2 (D, I, *n* = 7), 6X-W6 (E, J, *n* = 6) rats. Data are presented as mean ± SD.

**FIGURE 8:**
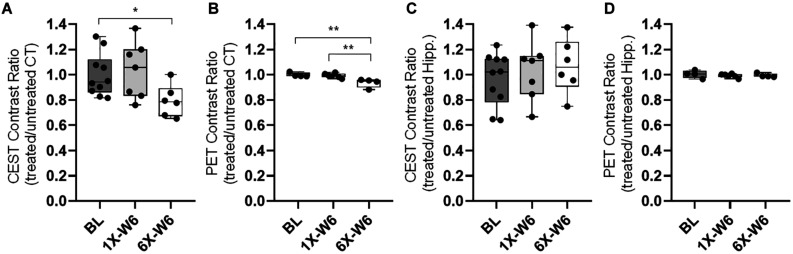
**(A)** The quantification of glucoCEST shows significantly decreased contrast ratios (treated/untreated) in the cortex of the Group 2 rats compared to that of the baseline (BL) control rats. **(B)** The FDG-PET quantification shows significantly decreased SUV ratios (treated/untreated) in the cortex of the Group 2 rats sonicated six times (6X-W6) compared to control animals and Group 1 rats sonicated once (1X-W6). No significant difference is seen in the hippocampus in the **(C)** glucoCEST and (d) FDG-PET data. *p < 0.05, **p < 0.01 vs. BL, Kruskal–Wallis one-way ANOVA, Dunn’s test (Group 1, *n* = 7; Group 2, *n* = 6).

No obvious difference was seen in the glucoCEST-weighted images between the treated and untreated cortex and hippocampus in Group 1 rats at either 2 or 6 week time points following a single pFUS+MB treatment (Figure 5). There was significant difference in the glucoCEST contrast ratios between the treated and untreated cortex of the baseline control rats, rats sonicated once and imaged in week 6, and rats sonicated six times and imaged in week 6 [H_(3)_ = 6.360, *p* < 0.05] (Figure 8A). The *post-hoc* tests showed the differences were between the animals of unsonicated baseline (0.998 ± 0.169) and the animals sonicated six times and imaged in week 6 (0.793 ± 0.126, *p* < 0.05). No significant difference was noted in the hippocampus. Longitudinally, in the Group 2 animals that had weekly sonication, there was a decrease trend in the contrast ratios in the cortex and hippocampus between week 2 and week 6 (Supplementary Figure S2). These changes were not significant in either of the location. Figures 6, 7 shows the group averaged Z-spectra and MTR_asym_ of the treated and untreated cortex and hippocampus.

### FDG-PET

FDG-PET studies were performed in the baseline control rats, Group 1 and Group 2 rats at week 6. There was a significant difference in the cortex among the three groups [H_(3)_ = 8.095, *p* < 0.01] (Figures 5, 8). The *post-hoc* tests showed significant decreases in the ratios between the unsonicated rats (1.00 ± 0.02) and Group 2 rats (0.94 ± 0.04, *p* < 0.01), and between Groups 1 (0.99 ± 0.02) and Group 2 rats (0.94 ± 0.04, *p* < 0.01) (Figure 8B). No significant difference was seen in the hippocampus among the three groups (Figure 8D). The quantifications of glucoCEST and FDG-PET results are also listed in the Supplementary Table S2.

### Histology

**FIGURE 9:**
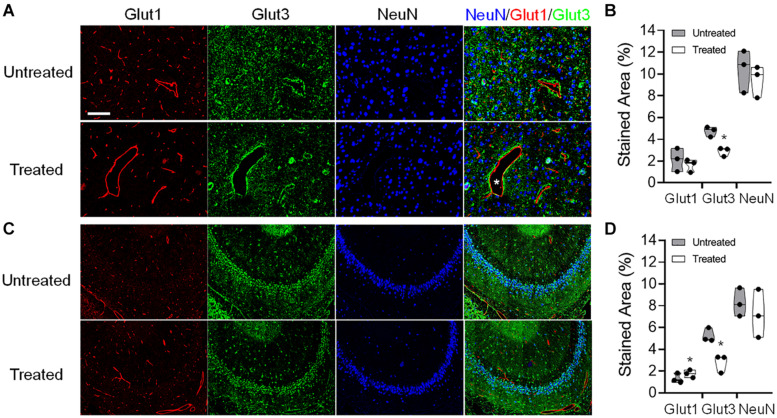
Representative immunohistochemistry (IHC) staining of the endothelial glucose transporter 1 (GLUT1), neuronal glucose transporter 3 (GLUT3), and neuronal nuclei (NeuN) of a Group 2 rat brain that was obtained at week 7 after six pFUS+MB treatments. Dilated vessels were found in the treated hemisphere (asterisk). The IHC images were acquired and quantified from the **(A,B)** cortex and **(C,D)** hippocampus. *p < 0.05, **p < 0.01 vs. contralateral untreated region, paired *t*-test (*n* = 3/stain/group).

Histological evaluations were performed on the tissues harvested in week 7 after a single or six weekly pFUS+MB exposures (Figure 9). Group 1 rats demonstrated no difference in GLUT1 or GLUT3 expression on the immunofluorescent staining in either sonicated region. In Group 2 rats, increased GLUT1 was detected on the endothelium of the dilated vessels in the treated hippocampus [*t*_(__2)_ = 4.483, *p* < 0.05]. Compared to a homogenous expression in the contralateral parenchyma, the GLUT1 expression was demonstrated as a scattered depleted pattern on the microvasculature in the sonicated sites. Neuronal GLUT3 expression was significantly decreased in the sonicated cortex [*t*_(__2)_ = 4.726, *p* < 0.05] and hippocampus [*t*_(__2)_ = 5.334, *p* < 0.05] as compared to the contralateral regions. No significant change was observed in the NeuN staining for neurons.

## Discussion

The major focus of this study was to use advanced MRI techniques and FDG-PET to evaluate the rat brain following single or 6 weekly pFUS+MB induced BBBO sessions over time. The majority of rats in Groups 1 and 2 had clear evidence of morphological changes on high-resolution T2^∗^w and T2w along with changes in FA on DTI images on MRI at 9.4 Tesla. In the animals receiving multiple weekly pFUS+MB, there was a decrease in the image contrast on the glucoCEST-weighted imaging and FDG-PET in the cortex when compared to unsonicated animals or the Group 1 rats sonicated once in week 6.

Various pFUS+MB protocols have been used in both experimental and clinical studies that result in BBBO, facilitating the delivery of neurotherapeutics to targeted regions in the brain and potentially enhancing neurological outcomes (Kroll and Neuwelt, 1998; Hynynen et al., 2001; McDannold et al., 2008, 2017; Neuwelt et al., 2008; Gabathuler, 2010; Burgess et al., 2014; Chai et al., 2014; Horodyckid et al., 2017; Kovacs et al., 2017b, 2018a; McMahon et al., 2017; Wu et al., 2017). The interaction of the pFUS with the MB confined to the vascular space leads to stable cavitation and transient BBBO via stretching of endothelial cells coupled with the induced expression of proinflammatory molecular proteins from the neurovascular unit and alterations in TJP expression (Sheikov et al., 2008; Aryal et al., 2015; Downs et al., 2015b; Kovacs et al., 2017b; McMahon and Hynynen, 2017; McMahon et al., 2017). The evaluation of the sonicated brain by MRI has usually been limited to GdT1w, T2w, T2^∗^w or susceptibility-weighted imaging along with quantitative metrics determining gadolinium leakage (Aryal et al., 2015; Downs et al., 2015b; Wu et al., 2017) and has rarely shown damage (Kovacs et al., 2018b). Conventional imaging techniques used following pFUS+MB BBBO are usually performed with relatively large voxels (>250 μm in-plane resolution and slices 0.8–1.5 mm) that are then correlated to pathological changes in the parenchyma (Treat et al., 2007, 2012; Chai et al., 2014; McDannold et al., 2016; Aryal et al., 2017; McMahon et al., 2017; O’Reilly et al., 2017; Kovacs et al., 2018b). In most of these imaging studies, pathological changes in the sonicated parenchyma could be missed especially when MRI is acquired at 3 T in small animal models (Treat et al., 2007; Marquet et al., 2011).

In the current study, MRI scans at 9.4 T were performed with in plane resolution of 200 × 200 μm^2^ and 500 μm thickness for T2w images and T2^∗^w and DTI images were performed at 200 μm thickness, thereby limiting partial volume effects and increasing the conspicuity of changes in signal intensity in the sonicated cortex and hippocampus compared to contralateral brain. T2w and T2^∗^w images acquired 1-day post-pFUS+MB did not reveal any abnormalities, however after two weeks, 71.4% of the rats had hypointense voxels in T2^∗^w images of the targeted cortex or hippocampus. It has recently been shown that 4 days following pFUS+MB in the rat, T2^∗^ hypo-intensities were detected suggesting vascular compromise in area of sonication (McMahon et al., 2017). Further investigation into the changes in vascular integrity would contribute to the evolution of pathological changes over time following pFUS+MB BBBO. Multiple weekly pFUS in the rat performed at higher PNP with Definity at 10 μL/kg diluted 10× demonstrated evidence of hypointense voxels on T2w image at 3 T associated with microhemorrhages (Kobus et al., 2016). In comparison, repeated pFUS+MB BBBO in non-human primates over 20 months did not show evidence of long term damage or hypointense voxels on SWI studies at 3 T (Downs et al., 2015b). It has recently been reported that T2w and T2^∗^w pathological changes were conspicuous at 3 T following 2 weekly pFUS+MB and there was a quantitative increase in numbers of voxels with lower T2^∗^ values following six sonications (Kovacs et al., 2018b). The current study demonstrated similar pathological changes observed at 9.4 T on T2w and T2^∗^w imaging following 2 and 6 weekly sonication to induce BBBO. The T2^∗^ pathology presumably due to microhemorrhages would evolve over time and either be metabolized by microglia and disappear or result in hemosiderin deposition in the brain and remain as an abnormality on T2^∗^ w images. The microhemorrhage changes in the rat brain following pFUS would be tracked on MRI similar to what is observed with ischemic disease, stroke or chronic traumatic encephalopathy in the rat.

There have been no reports to date in the rat employing DTI and CEST MRI to evaluate morphological and metabolic changes in targeted area in the brain. pFUS+MB induced changes on the FA maps of the targeted brain at 2 or 6 weeks compared to the contralateral brain. In comparison to the Group 1 rats, Group 2 rats exhibited decreased FA and AD in the sonicated external capsule that may reflect white matter damage, inflammation, neurite beading and disrupted glial cells (Budde and Frank, 2010; Tu et al., 2016). The increased FA in the sonicated cortex and hippocampus would suggest tissue damage in association with astrogliosis in the Group 2 rats (Budde et al., 2011). The decreases in RD in Group 2 rat cortex and hippocampus would be indicative of tissue swelling and/or increased cellularity, i.e., mononuclear cell infiltration and inflammation, in the sonicated regions, while the increased RD in the external capsule may be related to increased permeability or demyelination (Tu et al., 2014, 2016). For the 6 weekly pFUS+MB treated animals, there was greater significant T2^∗^ shortening in the cortex and hippocampus that could also contribute to the differences in the DTI metrics caused by local inhomogeneity, such as micro-hemorrhage and iron metabolites resulting in hyperintense regions on the FA maps compared to contralateral side. Further investigations are needed to determine the DTI metric changes immediately after sonication and how these changes relate to axonal integrity in the external capsule or neuronal damage within the parenchyma.

There have been few studies investigating glucose uptake or metabolism in normal brains by FDG-PET following BBBO by US combined with MB infusion (Yang et al., 2014; Horodyckid et al., 2017). Using a planar ultrasound transducer implanted in non-human primate skull with infusion of MB (SonoVue^TM^; Bracco, Milan, Italy) every 2 weeks, the authors reported BBBO but no evidence of change in glucose metabolism by FDG-PET (Horodyckid et al., 2017). In comparison ^18^F-FDG-PET performed immediately and 24 h after pFUS+MB showed decreased glucose uptake in the rat brain by inhibiting GLUT1 expression within the 24 h period of BBBO as documented by Evans blue extravasation into the parenchyma (Yang et al., 2014). The effect of pFUS+MB on glucose uptake was found to be transient and reversible after one sonication session. In the current study ^18^F-FDG-PET detected decreased FDG uptake and metabolism in rats receiving 6 weekly pFUS+MB treatments in the cortex but not at the level of the hippocampus. Changes in FDG uptake were not apparent in the treated brain 6 weeks after a single sonication. We also demonstrated that the glucoCEST-weighted images provided sufficient contrast reflective of decreased glucose levels in corresponding areas of decreased FDG uptake in Group 2 rats (Figures 5, 8). CEST-MRI has a voxel size 80–100 times smaller than PET scans by monitoring ^1^H - water ^1^H exchange on MRI without using radioisotope (Guivel-Scharen et al., 1998; Ward et al., 2000). The chemical exchange results in the transfer of magnetization from the small exchangeable proton pool (e.g., micromolar to millimolar range) to a much larger water proton pool (∼110 M) until a steady state is reached. The sensitivity of glucoCEST is higher than direct observation of the glucose through ^1^H-NMR because the proton signal is amplified via many exchange events during the extended saturation period of magnetization transfer pulses (Van Zijl and Yadav, 2011).

*In vivo* glucoCEST-weighted imaging has been shown to detect delayed hypo-metabolism in a rat brain following traumatic brain injury (Tu et al., 2018). The imaging contrast in glucoCEST-weighted imaging may be related to the steady-state glucose level in the brain parenchyma which has been previously validated by 2DG autoradiography. CEST data acquisition using low saturation power (1.5 μT) and short saturation duration (1 s) minimizes extensive direct saturation effect to enhance the sensitivity for detecting glucose in the rat brain. Although the endogenous glucoCEST contrast may also be affected by other hydroxyl metabolites such as myo-Inositol, deriving the glucoCEST-weighted contrast by integrating the MTR_asym_ areas specifically on the glucose chemical shifts at 1.2 ppm provides the sensitivity and specificity to detect changes of glucose level in the sonicated parenchyma (Figure 5; Chan et al., 2012; Tu et al., 2018). In this study, the IHC analysis suggests that the changes of the cerebral glucose level could be related to decreased neuronal transporter GLUT3, and the re-distributed vascular glucose transporter GLUT1 after multiple pFUS+MB BBBO sessions. While no significant loss of neuronal cells was seen in the NeuN staining, the decreased GLUT3 expression may be associated with the prolonged inflammatory responses, neurogenesis and neurodegenerative processes induced by repeated BBBO in the treated parenchyma (Kovacs et al., 2018b). A clear pattern of altered endothelial GLUT1 expression on the dilated vessels or on the contracted vessels may also decrease the glucose level in the sonicated brain (Figure 9). Further investigations are needed to evaluate the longer-term effects of pFUS+MB on endothelial GLUT1 and neuronal GLUT3 expression and whether the changes would return to control levels.

The recent progress in the pFUS+MB BBB research has advanced this novel technology to an encouraging direction for clinical application for drug delivery in the CNS diseases (NCT03321487, NCT02986932, NCT03608553, NCT03616860, NCT03739905, NCT03714243) (see text footnote 1). This contemporary technology continues to require optimization of the FUS parameters with the use of advanced passive cavitation detection feedback approaches to limit non-linear stable or inertial cavitation that could result in parenchymal damage or excessive neuroinflammation (Jones et al., 2018). The translational work would be to see if it will be possible to apply DTI and CEST, or a comparison to FDG-PET, for the pFUS+MB treatment in brain tumors or Alzheimer’s disease, and whether at higher resolution scans can detect subtle differences in glucose concentration which may reflect micrometastasis in the case of metastatic disease to the brain or glioblastoma multiforme spreading outside Gd enhanced area on FLAIR or T2w images. For Alzheimer’s disease, these imaging methods could be correlated to areas of hypoperfusion.

The current study employed a pFUS+MB protocol with nine 2-mm diameter focal spots in the left frontal cortex and four in the right hippocampus to provide an example of almost complete coverage in the hemisphere to induce BBBO and deliver neurotherapeutics or stimulate an immune response in the diseased brain (Kovacs et al., 2017b, 2018b). The experimental results may not be related directly to other pFUS+MB experiments to cause BBBO performed with different FUS parameters, targeting approaches or MB type, dose and infusion rates in relationship to initiating sonication (Kovacs et al., 2018a). Another limitation of this study is the lack of longer-term follow-up in animals that received 6 weekly sonications. It will be important to apply advanced imaging techniques to monitor changes in the brain following multiple sonication treatments to determine both morphological and metabolic responses in relationship to pathology and functional outcomes (Downs et al., 2015a; Kobus et al., 2016; Horodyckid et al., 2017; Kovacs et al., 2017a; O’Reilly et al., 2017; Silburt et al., 2017). Our findings suggest that the structural damage in the T2^∗^ and DTI may be more permanent, and the CEST and PET observation of the altered glucose levels may be transient and reversible in the longer term. On the detection of glucose utilization, the FDG-PET was more sensitive to detect the treatment effect with fewer data variations and higher significance. The glucoCEST data varied due to the less abundant endogenous glucose signals and the susceptibility to the imaging artifacts, including motion and field inhomogeneity. Since glucose signal at 1.2 ppm is close to the water signal, large saturation pulses may diminish the glucose signals due to the direct saturation effect. In order to observe the glucose signal, our previous study uses relatively small saturation intensity (1.5 μT and 1 s) for acquiring glucoCEST data on the 9.4 T for the rat brain (Tu et al., 2018). Further study is required to investigate the CEST imaging parameters for optimizing endogenous glucoCEST. Furthermore, the glucoCEST weighted image was generated by integrating the MTR_asym_ centered in 1.2 ppm, ranging from 0.7 to 1.7 ppm. The MTR_asym_ at this range may contain interferences from other metabolite signals, including creatine centering in 1.95 ppm, myo-inositol in 0.6 ppm, and the upfield Nuclear Overhauser Enhancement (NOE). More sophisticated CEST contrast analysis, such as multi-pool Lorentzian fitting of the global Z-spectra (Desmond et al., 2014; Zhou et al., 2017), may enhance the specificity of glucose detection following pFUS+MB BBBO in the brain. GlucoCEST imaged with exogenous glucose injection should improve the sensitivity to detect the subtle treatment effect of the pFUS+MB. However, the exogenous glucoCEST may be predominantly affected by the increased vessel permeability and glucose agent leakage from the pFUS+MB induced BBBO, which may not be related to the static glucose levels in the treated brain. The feasibility of applying exogenous glucoCEST in the case of pFUS+MB BBBO would require further investigations.

## Conclusion

This study describes the monitoring of the long-term effect using conventional and advanced imaging techniques such as DTI and CEST imaging following a single or multiple weekly pFUS+MB BBBO. The findings suggest the importance of using these imaging methods for monitoring the late effects in the brain tissue and be used for further investigations to evaluate the changes in the brain following low pressure pFUS+MB treatment.

## Data Availability Statement

All datasets generated for this study are included in the article/Supplementary Material.

## Ethics Statement

The animal study was reviewed and approved by the National Institutes of Health.

## Author Contributions

JF, T-WT, and ZK: conceptualization. T-WT, ZK, MS, JW, GP, WR, DH, and JF: methodology. T-WT, ZK, MS, JW, and GP: formal analysis. DH and JF: resources. T-WT and JF: writing – original draft. T-WT, ZK, DH, and JF: writing – review and editing. T-WT, ZK, and MS: visualization. DH and JF: supervision. T-WT, DH, and JF: funding acquisition. All authors had full access to all the data in the study and take responsibility for the integrity of the data and the accuracy of the data analysis.

## Conflict of Interest

The authors declare that the research was conducted in the absence of any commercial or financial relationships that could be construed as a potential conflict of interest.

## Footnotes

1 clinicaltrials.gov
