# Supplementary material for: Diffusion Tensor Imaging and Chemical Exchange Saturation Transfer MRI Evaluation on the Long-Term Effects of Pulsed Focused Ultrasound and Microbubbles Blood Brain Barrier Opening in the Rat
Source: Front Neurosci. 2020 Aug 25;14:908. doi: 10.3389/fnins.2020.00908 (PMC7478124; doi:10.3389/fnins.2020.00908)
Supplement: Supplementary file 1 [file Data_sheet_1.PDF]

SUPPORTING INFORMATION

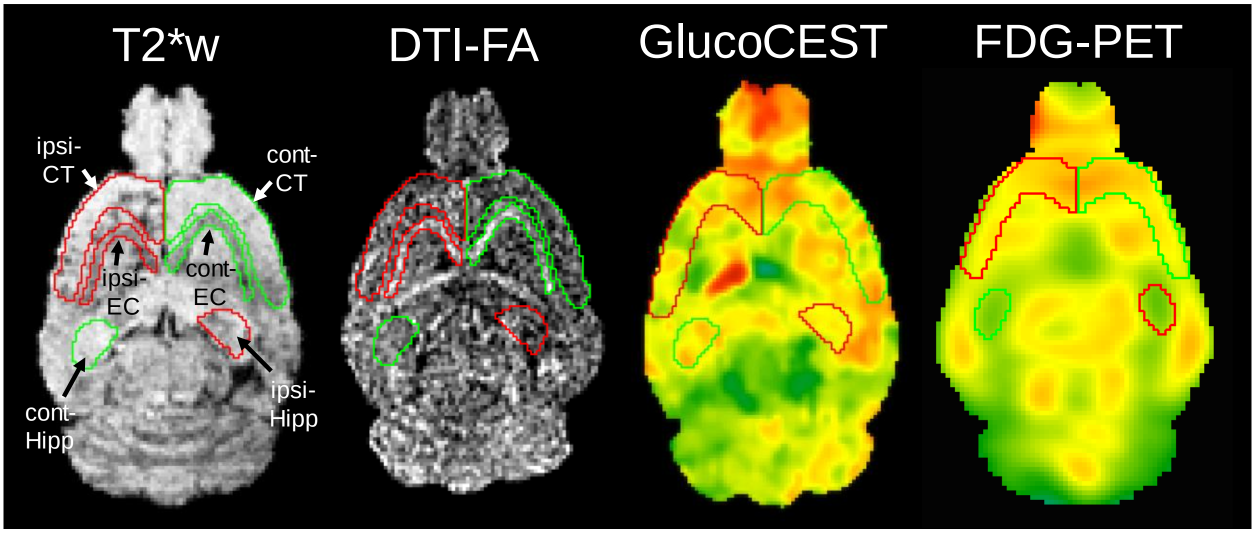

Supplementary Figure S1. The image analysis was performed through the regions of interest (ROIs) encompassing the ipsilateral (ipsi-, red ROIs) treated and contralateral (cont-, green ROIs) untreated cortex (CT), hippocampus (Hipp), and external capsule (EC).

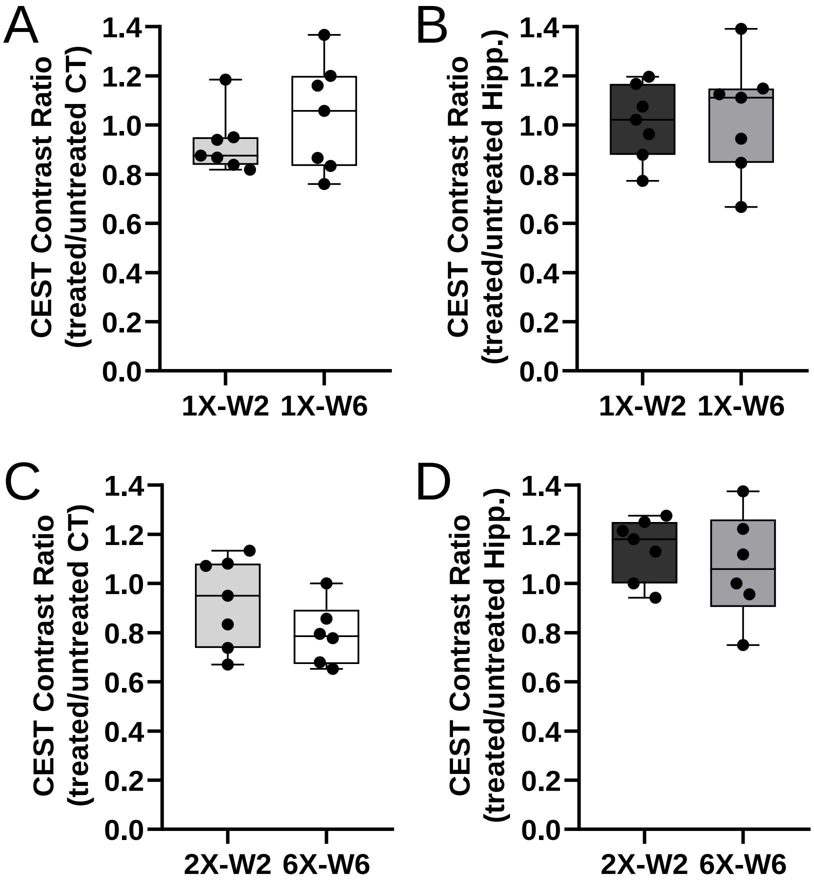

Supplementary Figure S2. The longitudinal glucoCEST showed a slight increase of the contrast ratios (treated/untreated) in the (A) cortex and (B) hippocampus of the Group 1 rats between the week 2 and week 6 scans, while that of the Group 2 rats exhibited a decrease trend (C, D). Kruskal–Wallis one-way ANOVA, Dunn's test (Group 1, n = 7/each time; Group 2, n = 7 for week 2, n=6 for week 6).

Supplementary Table S1. 9.4T imaging data acquired in week 6 brains of Group 1 rats with a single pFUS+MB sonication and Group 2 rats with 6 weekly sonications. Data are reported as mean ± standard deviation. * p<0.05, ** p<0.01 by paired t-test between the ipsi-lateral treated and contra-lateral untreated region. (Group 1, n = 7; Group 2, n = 6).

Group	Region	Hemisphere	T2* (ms)	FA	MD ($\mu m^2/ms$)	AD ($\mu m^2/ms$)	RD ($\mu m^2/ms$)
Single pFUS+MB (Group 1)	CT	Untreated	48.86±1.97	0.44±0.03	0.76±0.03	1.11±0.04	0.58±0.04
		Treated	45.90±5.44	0.42±0.04	0.74±0.02	1.08±0.04	0.58±0.02
	EC	Untreated	28.96±2.77	0.78±0.03	0.61±0.05	1.30±0.13	0.28±0.04
		Treated	28.45±1.21	0.75±0.03	0.66±0.05	1.34±0.08	0.32±0.04*
	Hipp	Untreated	50.19±2.55	0.40±0.04	0.76±0.04	1.10±0.07	0.60±0.04
		Treated	35.23±4.43**	0.42±0.06	0.78±0.04	1.14±0.09	0.61±0.06
Weekly pFUS+MB (Group 2)	CT	Untreated	44.84±3.55	0.51±0.07	0.64±0.05	1.00±0.04	0.46±0.07
		Treated	34.42±7.08**	0.57±0.10*	0.62±0.07	1.00±0.09**	0.42±0.09
	EC	Untreated	29.46±2.86	0.78±0.02	0.64±0.07	1.33±0.17	0.29±0.03
		Treated	27.18±2.42	0.70±0.01**	0.64±0.05	1.21±0.11*	0.35±0.02*
	Hipp	Untreated	46.98±3.81	0.45±0.09	0.71±0.06	1.05±0.09	0.55±0.07
		Treated	30.76±4.96**	0.56±0.10**	0.66±0.07	1.07±0.05*	0.45±0.10*

Supplementary Table S2. GlucoCEST and FDG-PET data acquired in the baseline control (BL), week 6 brains of Group 1 rats with a single pFUS+MB sonication (1X-W6), and Group 2 rats with 6 weekly sonications. Data are listed as mean ± standard deviation. * p<0.05, ** p<0.01, compared to BL; † p<0.01, compared to 6X-W6, by Kruskal–Wallis one-way ANOVA, Dunn's test (Group 1, n = 7; Group 2, n = 6).

Method	Region	BL	1X-W6	6X-W6
GlucoCEST	CT	1.00±0.17	1.03±0.22	0.79±0.13*
	Hipp	0.97±0.21	1.03±0.24	1.07±0.22
FDG-PET	CT	1.00±0.02	0.99±0.02†	0.94±0.04**
	Hipp	1.00±0.03	0.99±0.01	0.99±0.02
